# Comparative analysis of opioid use in sickle cell crisis in an urban facility in Ghana

**DOI:** 10.1002/jha2.704

**Published:** 2023-05-11

**Authors:** Marian Opoku‐Agyakwa, Henry J. Lawson, Edeghonghon Olayemi

**Affiliations:** ^1^ Family Medicine Department University of Ghana Medical Centre Accra Ghana; ^2^ Family Medicine Unit Community Health Department University of Ghana Medical School Accra Ghana; ^3^ Haematology Department University of Ghana Medical School Accra Ghana

**Keywords:** opioid, pain control, sickle cell disease, sustained pain response, vaso‐occlusive crisis

## Abstract

Vaso‐occlusive crises (VOC) is common and opioids are the treatment of choice.This study compared parenteral pethidine and morphine in the elimination/reduction of pain in acute VOC to tolerable levels. This open‐label randomized study compared intravenous morphine 5 mg 4 hourly to intramuscular pethidine 75 mg 4 hourly. Eighty‐two consenting adult sickle cell disease participants were recruited from the Korle‐Bu Polyclinic. (Forty‐one participants in each arm). There were 42 male and 40 female participants. Median age was 25 years. Pethidine participants totalling 31.7% (13/41) and 53.7% (22/41) in the morphine arm had a sustained response within 6 h, *p* = 0.027. In the pethidine and morphine arms 60.0% (24/40) and 62.5% (25/40) of participants respectively achieved adequate pain control within 72 h of initiating therapy, *p* = 0.296. Most participants, 96.3% (79/82) had no side effects to opioids. The commonest side effects were generalized pruritus, nausea and vomiting, and headaches. More pethidine than morphine participants experienced side effects 29.3% and 22.0% respectively; *p* = 0.448. In conclusion, more morphine participants achieved a sustained pain response compared to the pethidine participants. There was no difference in the tolerability and side effect profile of the opioids. No participant experienced respiratory suppression.

## 1 INTRODUCTION

Scientific background: Sickle cell disease (SCD) is a chronic debilitating, inherited haemoglobinopathy common in sub‐Saharan Africa [1] due to inheritance of the Hb S gene along with another abnormal haemoglobins [2]. The specific genotype largely determines the severity of the disease.

Annually, about 300,000 infants are born with sickle cell anaemia with almost 80% in sub‐Saharan Africa; the number is projected to rise to approximately 400,000 by 2050 [3].

Acute painful episodes (due to vaso‐occlusion, infarcted tissue, and release of inflammatory mediators) [4] is the principal symptom of SCD and the most common reason for acute admission. Three or more severe pain crises in a year imply severe disease [5]. Frequent painful crises are associated with a reduced quality of life and an increased risk of death [6]. There are several guidelines for the management of acute pain crises; however, there is no consensus on the single best protocol [3]. Largely, pethidine and morphine are the most used opioids for initial analgesia in severe SCD pain crisis [7].

Unfortunately, certain barriers limit the effective use of these opioids. First, there are concerns from healthcare workers, family members and even the patients related to the possible side effects, the development of tolerance associated with chronic use, and the risk of dependence/addiction. In the USA, pethidine and morphine are categorized as schedule II for controlled substances. This classification indicates their level of addiction potential [8].

The acute pain of SCD is sometimes difficult to treat [9]. There are different views on the treatment of the pain, which have to do with the suitability of opioids, and which is preferred, the efficacy of parenteral administration, and the risk of dependence on opioids.

This study compared parenteral pethidine and morphine in the elimination/reduction of the acute pain of vaso‐occlusive crises (VOC) in adult SCD patients. We tested the hypothesis ‘There is no difference between morphine and pethidine in time to achieving sustained pain relief and their safety profiles in adult SCD patients with an acute pain crisis’.

## 2 METHODS

The study was an open randomized comparative study of parenteral Morphine and Pethidine in adult (18 years and above) Sickle Cell Disease patients presenting to the Korle Bu Polyclinic. Previous studies suggest that morphine is more effective in relieving pain crises than pethidine [9, 10].

All consenting adult sickle cell patients in acute pain crisis, with a minimum pain score of 7 on the McCaffery et al Pain Scale [13] during the period of the study were included. The exclusion criteria included a history of opioid allergy or adverse effect to opioid use, urine dip‐stix of greater than 2+ proteinuria, history of moderate to severe liver disease, or moderate to severe jaundice, pregnant women, patients with cognitive impairment, uncontrolled seizure disorder and uncontrolled asthma. One arm received 5 mg of intravenous (IV) Morphine Sulphate Fresenius Preservative Free (PF) 4 hourly, while the other arm received intramuscular Pethidine Hydrochloride British Pharmacopoeia in alternate glutei muscles at a dose of 75 mg 4 hourly. Every participant got oral paracetamol 1 g thrice daily, bisacodyl 5 mg at night, IV metoclopramide 10 mg thrice daily and 2–3 L Dextrose/Saline daily. Other medications were given as indicated, and laboratory investigations ran. The data abstraction form containing the demographics, SCD phenotype, past medical history, and drug history was filled for each participant. Hourly pain scoring was done and documented except when the patient was asleep, in which case same was documented. All adverse drug events and side effects of medication were also documented, and a study physician informed. Treatment was implemented as deemed appropriate. The research assistants were the clinic nurses since they have the technical skill of administering medication and identifying their side effects.

**All patients received the standard of care that sickle cell patients receive at the study site**. The primary outcome of the study was the proportion of participants in each arm who achieved pain control. That is, the proportion of participants whose admission pain reduced from a rating of at least seven to a level three or below (and remained so continuously for 24 h) within 72 h following the commencement of the analgesia. Secondary outcome measures were, the time taken for the initial pain to reduce by a margin of at least 2 within a 6‐h period (even if the pain went up thereafter), as well as the incidence of adverse events in the two treatment groups. The time interval for the complete resolution of crisis between the two arms was measured.

Operative definitions derived for the purposes of this study. They were: (1) duration for initial response: defined as the time taken for a reduction in pain score of at least 2 points, even if pain score went up later; (2) duration for sustained response: defined as the time taken for a decrease in the pain score of 2 or more points and continuing as such (the pain does not go up again); (3) duration for pain control: defined as the time taken for the pain to decrease from at least 7 to 3 or below and remain so continuously for a minimum of 24 h.

The study was approved by Korle Bu Teaching Hospital Institutional Review Board (KBTH‐ IRB/004/2015) and registered with the Pan African Clinical Trial Registry database (PACTR201812917283565). Unpaired Student's *t*‐test was used to compare the pain duration between pethidine and morphine. The chi‐squared test was used to compare the tolerability levels and the side effect profile between the two analgesics. Regression analysis was carried out to determine the effect of demographic characteristics on pain control and to account for possible confounders. Pain control indices were used as dependent variables whilst the demographic characteristics and the presenting pain grade were used as independent or explanatory variables in the regression model. *p*‐Values were considered significant if less than 0.05 at a confidence interval of 95% (Table 1).

**TABLE 1:** Pain grade interpretation.

Pain grade	Interpretation
0	No pain
1–3	Mild pain
4–6	Moderate pain
7–10	Severe pain

## 3 RESULTS

There were a total of 82 participants, aged 18 to 47 years. The median age 25.0 years (SD‐6.3years) and 42 (51.2%) were males. Sixty‐seven (81.7%) of the participants were SS phenotype; the remainder were SC. The proportions of haemoglobin phenotype in the age categories did not differ significantly from each other at the 0.05 level (Table 2).

**TABLE 2:** Comparison of analgesia and duration of pain control within 72 h (*n* = 80).

Analgesia	Controlled (%)	Uncontrolled (%)	*χ* ^2^	*p*‐Value	Total	Median (hours)	*p*‐Value
Pethidine	24 (60.0)	16 (40.0)	0.005	1.000	40	66.7	0.296
Morphine	25 (62.5)	15 (37.5)			40	72.5	
Total	49 (61.3)	31 (38.8)			80		

In the pethidine arm, 24 (60.0%) of the 40 participants had their pain controlled within 72 h of initiating therapy. In the morphine arm, 25 (62.5%) of the 40 participants had their pain controlled within 72 h of initiating therapy. The difference in duration for pain control between the two groups was not statistically significant, *p*‐value = 0.296.

Twenty‐two participants in the morphine arm achieved a sustained response within 6 h of initiating treatment, and 15 between 6 to 24 h. In the pethidine arm, 13 participants achieved a sustained response within 6 h of treatment, and 19 between 6 and 24 h.

Twenty‐two participants (52.4%) in the morphine arm and 13 (31.7%) in the pethidine arm, achieved a sustained response within 6 h of commencing treatment. The duration for sustained response showed a statistically significant difference between the two opioids (*p* = 0.027) with morphine significantly faster than pethidine.

A total of 23 side effects were experienced in 21 participants. More pethidine participants experienced side effects 12 (29.3%) compared to those in the morphine arm 9 (22.0%) (Table 3). However, the difference was not statistically significant, *p* = 0.448.

**TABLE 3:** Comparison of analgesia with duration for sustained response (*n* = 82).

Analgesia	Within 6 h (%)	Greater than 6 h (%)	χ^2^	*p*‐Value	Total	Median (hours)	*p*‐Value
Pethidine	13 (31.7%)	28 (68.3%)	3.1.903	0.07408	41 (100.0%)	9.6	0.027
Morphine	22 (52.4%)	20 (27.6%)			41 (100.0%)	17.4	
Total	35 (42.7%)	47 (57.3%)			82 (100%)		

*Mann–Whitney U.

The commonest side effects were generalized pruritus, nausea and vomiting and headaches (Table 4).

**TABLE 4:** Prevalence of the side effects of the analgesia (*n* = 82).

Analgesia	Side effects		*p*‐Value	Total
	Yes (%)	No (%)		
Morphine	9 (22.0)	32 (78.0)	0.448	41
Pethidine	12 (29.3)	29 (70.7)		41
Total	21	61		82

*Note*: OR = 0.680; 95% CI = 0.250–1.847.

Five patients had side effects of various combinations, four in the pethidine arm of the study and only one participant in the morphine arm. There were no significant differences between the two analgesics in terms of whether an individual side effect was observed or not, *p* = 0.370; neither was there any difference in what the specific type of side effect experienced was; *p* = 0.745. The side effect profiles of the two groups didn't differ significantly from each other. At an alpha level of 0.05, and df = 5, the Pearson's chi square, *χ* ^2^ = 6.146. None of the participants experienced features of Central Nervous System toxicity or respiratory depression.

## 4 DISCUSSION

The finding in this study of achieving pain control within 72 h in 60.0% of the participants in the pethidine arm and in 62.5% of the morphine participants, *p* = 0.296, is similar to a study by Charles et al.; where the median length of hospital stay in patients with uncomplicated VOC was 4 days [14]. Unlike the present study though, Charles et al. followed the 2003 BSH guidelines, by adding a tapering dose of opiate and NSAIDs to the treatment when the patient was pain‐free (Table 5).

**TABLE 5:** A breakdown of the side effects experienced in the morphine and pethidine arms respectively (*n* = 23).

Side effect	Morphine (%)	Pethidine (%)	*χ* ^2^	Total
Pruritus	3 (42.9)	4 (57.1)		7
Nausea/ Vomiting	3 (50)	3 (50)		6
Headaches	1 (20)	4 (80)		5
Dizziness/Drowsiness	0 (0)	2 (100)	6.146	2
**Unarousable**	2 (100)	0 (0)		2
Seizures	0 (0)	1 (100)		1
Total	9	14		23

Similar pain control effect with the analgesia indicates that acceptable pain control within the stipulated duration was observed in almost equal numbers in both study arms. This shows that the two analgesics are of comparable effectiveness in at least 60% of patients in severe pain from VOC. Resulting in a significant resolution of their pain within 72 h and a possible return to near normal activities soon thereafter.

Sustained response was observed within 6 h of starting treatment in 35 (42.7%) of the participants; 13 (32.5%) of the pethidine participants compared with 22 (52.4%) of the morphine participants (*p* **= 0.027)**. This shows that participants in the morphine arm experienced a faster initial decline in their pain grade compared with the pethidine participants.

It is interesting to note that the dose of analgesia had to be increased in more participants in the morphine arm, nine of 13 participants (69.2%), compared to pethidine, four of 13 participants (30.8%), to achieve a sustained pain response after the maximum allowable duration of 24 h. According to Bryant and Knight (2010), the average plasma half‐life of morphine is 2–3 h with the effect of a parenteral dose lasting for 4–6 h [14]. On the other hand, pethidine has a plasma half‐ life of 3–5 h and useful analgesia lasts between 2–4 h after parenteral administration. These facts imply that at the time of administering the next dose of parenteral analgesia, there is a lot more morphine still in circulation from the previous dose, compared to pethidine. The succeeding doses would add‐on to what is already in circulation and hence morphine should generally give a more rapid analgesic response than pethidine. Inter‐individual variability in the pharmaco‐dynamic parameters of morphine would explain the variability in pain intensity between patients and the dose of morphine needed to treat pain [15].

In this study, side effects were experienced 23 times affecting 21 participants (25.6%), 14 (60.9%) in the pethidine arm and 9 (39.1%) in the morphine arm. The side effects manifested were pruritus, nausea/vomiting, headaches, dizziness/drowsiness and seizures. There were no significant differences between the two analgesics in terms of whether a side effect was observed or not, *p* = 0.448. Neither was there any difference in what the specific type of side effect experienced was; *p* = 0.292.

Benyamin et al. found constipation and nausea to be the most common adverse effects, and this led to dropouts in controlled trials [16]. This observation partially conformed to the current study, but there were no dropouts. Another study whose findings somewhat conformed to the current study was the Huse et al. study, which found out that the most common adverse events were nausea (33% opioid versus 9% control) and constipation (33% opioid versus 10% control), followed by drowsiness (29% opioid versus 6% control) and vomiting (15% opioid versus 3% control) [17]. However, the findings of the current study were in disparity to that of Dellemijn et al. in which the researchers reported a high incidence of sweating, anorexia, and clouded vision [18].

Generally, respiratory depression and death are the most feared complications of opioid use. In this study, none of the participants experienced respiratory depression. This observation may have been due to the strict adherence to the study protocol as well as the guidelines of opioid use. Effective patient compliance with appropriate medical treatment programs is needed to prevent rare but life‐threatening adverse events [16].

For 79 (96.3%) of the participants, there were either no side effects 61 (74.4%) or manageable side effects, 18 (22.0%) to the analgesics and hence the opioids were not discontinued. Oral cetirizine 10 mg nocte was given for the pruritus, while the dose of IV metoclopramide was increased to 10 mg 6 hourly for those with nausea and vomiting. Oral paracetamol was increased to 1 g four times a day for one patient who had persistent severe headaches. The headaches were otherwise transient. The dizziness/drowsiness resolved without any intervention. This finding of the current study is supported by Smith who suggested that when these side effects occur, they are largely manageable [19]. In the Trinidad and Tobago study also, one of 82 admissions had an episode of seizure, but the authors could not exactly attribute it to the pethidine given [14].

For the other three participants in the current study, their analgesia had to be discontinued while in the study because the side effects were not tolerable. Two were in the morphine arm and the third in the pethidine arm. Both morphine participants developed acute chest syndrome and became **unarousable**. One of the morphine participants had her analgesia restarted when her sedation improved, and she began to experience significant pain. She later recovered and was discharged home. The other morphine participant got admitted to the Intensive Care Unit and died 16 days thereafter from multiple organ failure.

The pethidine participant had seizures and died 36 h after stopping the analgesia, and 12 h after an emergency dialysis was done for acute kidney injury perhaps secondary to hypovolaemia from vomiting and diarrhoea. He presented 7 days after his symptoms started.

As alluded to by Niscola et al., pethidine has a plasma half‐life of 3–5 h. However, the active metabolite norpethidine with a half‐life 15–40 h accumulates particularly in patients with renal failure. This may lead to potentially serious adverse effects including tremor, myoclonus, delirium, and seizures [2], probably manifested by the patient in the current study.

It is reassuring to observe that the two analgesics have comparable side effect profiles managed with similar interventions. Even more, these side effect profiles are generally safe and largely fleeting.

The advantage of combining paracetamol with opioids is the dual analgesic effect it provides. However, the combination is potentially hepatic toxicity [22]. In this study, a maximum dose of 3‐g paracetamol daily was used.

## 5 CONCLUSION

More morphine participants achieved a sustained pain response during compared to the pethidine participants. This difference was statistically significant. There was no significant difference in the effectiveness of pethidine and morphine in the reduction of acute pain in SCD patients within the first 72 h of initiating analgesia. Finally, there was no difference in the tolerability and side effect profile of pethidine in comparison to morphine in SCD patients presenting in VOC.

## AUTHOR CONTRIBUTIONS

*Conception and Study Design*: M O‐A and HL. *Data Collection*: M O‐A. *Data Analysis and Interpretation*: M O‐A, HL and EO. *Manuscript Drafting*: M O‐A. *Manuscript Revision*: EO and HL. *Guarantor of the Study*: M O‐A.

## CONFLICT OF INTEREST STATEMENT

The authors declare that they have no competing interests.

## FUNDING INFORMATION

Dr Marian Opoku‐Agyakwa

## ETHICS STATEMENT

The study was approved by Korle Bu Teaching Hospital Institutional Review Board (KBTH‐ IRB/004/2015) and registered with the Pan African Clinical Trial Registry database (PACTR201812917283565). All patients recruited provided written informed consent.

## CLINICAL TRIAL REGISTRATION

This clinical trial is registered with the Pan African Clinical Trial Database (PACTR201812917283565).

## Data Availability

The authors agree to the Data Sharing Policy of the journal.
